# Community Strains of Methicillin-Resistant *Staphylococcus aureus* as Potential Cause of Healthcare-associated Infections, Uruguay, 2002–2004

**DOI:** 10.3201/eid1408.071183

**Published:** 2008-08

**Authors:** Stephen R. Benoit, Concepción Estivariz, Cristina Mogdasy, Walter Pedreira, Antonio Galiana, Alvaro Galiana, Homero Bagnulo, Rachel Gorwitz, Gregory E. Fosheim, Linda K. McDougal, Daniel Jernigan

**Affiliations:** * Centers for Disease Control and Prevention, Atlanta, Georgia, USA; † Asociación Española, Montevideo, Uruguay; ‡ Hospital Maciel, Montevideo; § Hospital Pereira Rossell, Montevideo

**Keywords:** *Staphylococcus aureus*, CA-MRSA, HA-MRSA, antimicrobial resistance, healthcare-onset infections, Uruguay, research

## Abstract

Community-associated MRSA (CA-MRSA) strains have emerged in Uruguay. We reviewed *Staphylococcus aureus* isolates from a large healthcare facility in Montevideo (center A) and obtained information from 3 additional hospitals on patients infected with CA-MRSA. An infection was defined as healthcare-onset if the culture was obtained >48 hours after hospital admission. At center A, the proportion of *S. aureus* infections caused by CA-MRSA increased from 4% to 23% over 2 years; the proportion caused by healthcare-associated MRSA (HA-MRSA) decreased from 25% to 5%. Of 182 patients infected with CA-MRSA, 38 (21%) had healthcare-onset infections. Pulsed-field gel electrophoresis determined that 22 (92%) of 24 isolates were USA1100, a community strain. CA-MRSA has emerged in Uruguay and appears to have replaced HA-MRSA strains at 1 healthcare facility. In addition, CA-MRSA appears to cause healthcare-onset infections, a finding that emphasizes the need for infection control measures to prevent transmission within healthcare settings.

## Abstract 2

Community-associated MRSA appears to be replacing healthcare-associated MRSA strain types in at least 1 facility and is a cause of healthcare-onset infections.

Methicillin-resistant *Staphylococcus aureus* (MRSA) was recognized as a nosocomial pathogen in the 1960s and now represents a substantial proportion of *S. aureus* infections in inpatient and outpatient settings (*1*,*2*). Risk factors for healthcare-associated MRSA (HA-MRSA) are well defined and include hospitalization, surgery, dialysis, residence in a long-term care facility, and use of indwelling catheters or other percutaneous medical devices (*3*,*4*).

During the 1990s, MRSA emerged as a cause of infection among healthy persons in the community who had none of the above HA-MRSA risk factors (*5*–*10*). Community-associated MRSA (CA-MRSA) infections most commonly manifest as skin and soft tissue infections, but more invasive infections, including sepsis syndrome, necrotizing pneumonia, and fasciitis, also occur (*11*,*12*). Outbreaks of CA-MRSA infection have occurred among prisoners, sports participants, military recruits, and healthy full-term newborns (*7*–*9*). In a population-based study in Atlanta and Baltimore, the incidence of CA-MRSA infection was highest among children <2 years old (*11*). Factors that appear to facilitate transmission of CA-MRSA include frequent skin-to-skin contact, crowding, compromised skin integrity, sharing of potentially contaminated items, and lack of personal hygiene.

HA-MRSA and CA-MRSA possess resistance to β-lactam antimicrobial agents, conferred by the staphylococcal cassette chromosome (SCC) *mec* element (*13*). However, CA-MRSA strains are typically less resistant to non–-β-lactam antimicrobial agents (*14*). CA-MRSA strains almost invariably contain a particular SCC*mec* type (SCC*mec* IV, V, or VI), whereas HA-MRSA strains usually contain SCC*mec* types I, II, or III (*15*–*18*). CA-MRSA strains typically possess the Panton-Valentine leukocidin (PVL) toxin, which has been associated with skin abscesses and necrotizing pneumonia (*19*).

CA-MRSA has been reported worldwide, and several reports describe the entrance of CA-MRSA strain types into healthcare settings (*20*–*22*). However, few articles have documented CA-MRSA emergence in South America (*23*,*24*). The objectives of this investigation were to describe trends in *S. aureus* and MRSA infections in Uruguay, to explore transmission of these strains in healthcare settings, and to characterize CA-MRSA strains circulating in Uruguay.

## Methods

### Definitions

In this investigation, we defined a case of MRSA infection as illness compatible with staphylococcal disease in a patient from whom a strain of *S. aureus* resistant to oxacillin by disk diffusion was isolated from a clinically relevant site. Because it was suspected that community strains had entered the healthcare setting, epidemiologic risk factor data were not useful in distinguishing community versus healthcare strains. Therefore, microbiologic definitions were used. A MRSA isolate was considered to be an HA-MRSA strain if it was resistant to at least 2 of the following antimicrobial agents: trimethoprim/ sulfamethoxazole (TMP/SMX), ciprofloxacin, gentamicin, rifampin, and tetracycline. A MRSA isolate was considered to be a CA-MRSA strain if 1) antimicrobial susceptibility results were available for at least 2 of the following agents: TMP/SMX, ciprofloxacin, gentamicin, rifampin, tetracycline, and 2) the isolate was resistant to no more than 1 of the agents and was confirmed to be susceptible to at least 2 of these agents.

We considered an infection to be healthcare onset if the MRSA culture was obtained >48 hours after a patient was admitted to the hospital and the patient had no evidence of the infection at the time of admission. A MRSA culture obtained within 48 hours of hospital admission or evidence of infection on admission was considered an indication of a community-onset infection.

Skin disease was defined as a primary skin infection such as abscess, cellulitis, folliculitis, or a skin infection spreading to contiguous tissues. Surgical site infections (SSIs) were not considered to be skin disease.

### Assessment of Temporal Trends

To describe trends in *S. aureus* and MRSA infections, we reviewed laboratory records from August 2002 through July 2004 from a large healthcare facility (center A) that provided inpatient, outpatient, emergency, and long-term–care services to nearly 200,000 persons of all ages and socioeconomic levels. Reports of all *S. aureus* cultures, except nasal swabs (to exclude asymptomatic colonized patients), were included. Only the first culture was selected for each patient over the study period. Of the total number of *S. aureus* infections, the percentage due to MRSA was calculated for each quarter year of the study. Similarly, of the MRSA infections, the percentages caused by CA-MRSA and HA-MRSA were calculated for each quarter year. Chi-square tests for trend were calculated by using SAS version 9.1 software (SAS, Cary, NC, USA).

### Assessment of Healthcare Transmission

To explore transmission of CA-MRSA strains in hospitals and describe factors associated with transmission, we reviewed medical records of patients with CA-MRSA infections who were hospitalized between January 2003 and August 2004 at 4 facilities in Uruguay, centers A–D. Centers A and B were prepaid health maintenance organizations serving a heterogenous population of all ages and socioeconomic status. Centers C and D were large public referral hospitals serving a population of lower socioeconomic status. Center D was a pediatric hospital. At center A, we identified cases by reviewing laboratory records (including susceptibility data) of clinical *S. aureus* isolates (see Assessment of Temporal Trends). In the other 3 centers, microbiologists and infectious disease physicians provided a list of patients with MRSA infections. We identified the patients who met our microbiologic case definition by reviewing the patients’ laboratory records. Demographic and clinical data were abstracted from patient records by using a standardized form. Screening all patients for MRSA was not standard practice in any of the facilities included in this study.

Data collected included age, sex, location of residence (capital city of Montevideo vs. other locations), underlying medical conditions (chronic bronchitis, heart disease or stroke, liver or kidney disease, diabetes, HIV, AIDS, or history of immunosuppression or cancer), infection site (skin vs. non-skin), intensive care unit (ICU) admission, and onset of infection (hospital vs. community).

### Data Analysis

We performed multivariable analysis by using logistic regression to determine characteristics independently associated with healthcare-onset CA-MRSA strain type infections. Variable screening was performed by using univariate logistic regression with an α significance level of 0.25. Variables that met the screening criteria were entered in a multivariable model and retained with an α significance level of 0.05. Variables that failed to meet screening criteria were assessed as potential confounders by using β estimate changes of >15% as the criteria. We also assessed effect modification between facility and infection site, facility and age, and infection site and age.

### Laboratory Characterization of CA-MRSA Strains

Microbiologists and infectious disease physicians in Uruguay had a list of available isolates from patients infected with MRSA. We selected a convenience sample of 24 isolates from this list to obtain different clinical manifestations (skin and systemic infections), patient characteristics (pediatric and adult), treatment settings (inpatient and outpatient), healthcare institutions, and community and healthcare-onset disease. All isolates were from patients whose disease met our microbiologic definition of CA-MRSA disease. Isolates were sent to the Division of Healthcare Quality Promotion staphylococcal reference laboratory at the Centers for Disease Control and Prevention (CDC, Atlanta, GA, USA). After initially being screened for oxacillin resistance, isolates were tested for antimicrobial susceptibility by broth microdilution, according to the Clinical and Laboratory Standards Institute (*25*). In addition, antimicrobial-susceptibility disk tests were performed to study inducible clindamycin resistance (*25*). A PCR was used to characterize the SCC*mec* resistance complex and to detect the genes encoding the PVL cytotoxin, toxic shock syndrome toxin 1, and enterotoxins A–E and H (*15*,*26*). Pulsed-field gel electrophoresis (PFGE) was performed on restriction digests of chromosomal DNA (by using *Sma*I restriction endonuclease); gels were analyzed with BioNumerics software according to published criteria (*27*).

## Results

### Trends in *S. aureus* and MRSA Infections (center A)

**Figure 1:**
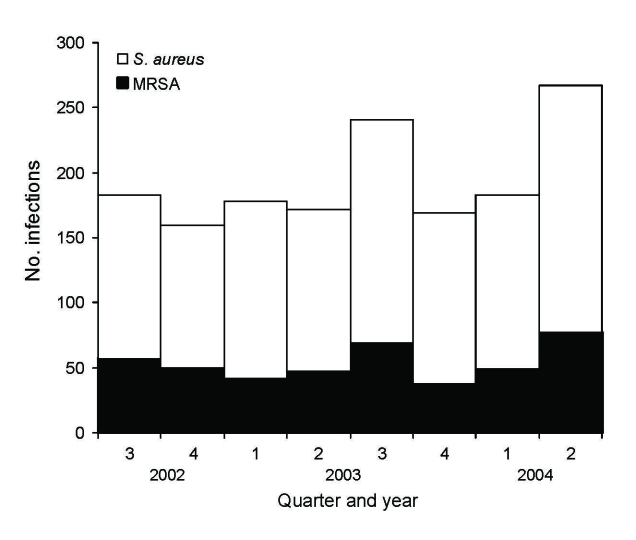
Number of *Staphylococcus aureus* and methicillin-resistant *S. aureus* (MRSA) infections by quarter and year, center A, August 2002–July 2004. N = 1,553.

**Figure 2:**
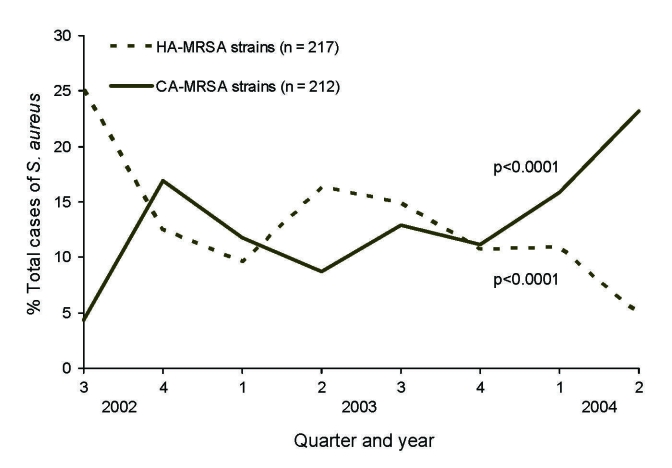
Proportion of *Staphylococcus aureus* due to community-associated methicillin-resistant *S. aureus* (CA-MRSA) infections and healthcare-associated MRSA (HA-MRSA) infections by quarter and year, center A, August 2002–July 2004.

Of 1,553 *S. aureus* infections at the health maintenance organization facility (center A), 42% were cultured in the hospital setting, 14% in the emergency department, 42% ambulatory care, and 2% from long-term-care service. The patients’ median age was 56 years, and 55% were male. The proportion of *S. aureus* infections caused by MRSA remained stable over the 2-year period (χ^2^ for trend p = 0.46), averaging 28% (Figure 1). CA-MRSA strains increased from 4% to 23% of all *S. aureus* infections (χ^2^ for trend p<0.0001) over the 2-year study period, whereas the proportion caused by HA-MRSA decreased from 25% to 5% (χ^2^ for trend p<0.0001) (Figure 2).

### CA-MRSA Infections among Patients Hospitalized in 4 Facilities (centers A–D)

Of the hospitalized patients with CA-MRSA in the 4 facilities, 59% were male, 80% were from Montevideo, and 29% had an underlying chronic medical condition (Table 1). Most infections were of the skin (63%), followed by respiratory infections (13%), bacteremias (9%), and SSIs (9%) (Table 2).

**Table 1:** Association of factors with healthcare- versus community-onset CA-MRSA, hospitalized patients, centers A–D, Uruguay, 2003–2004*†

Factors	Total no. (%), N = 182	Healthcare-onset, no. (%), n = 38	Community-onset, no. (%), n = 144	Univariate odds of healthcare-onset (95% CI)	Multivariate odds of healthcare-onset (95% CI)†
Age >18 y‡	79 (44)	33 (89)	46 (32)	17.4 (5.8–52.0)	4.8 (1.2–18.7)
Male	107 (59)	26 (68)	81 (56)	1.7 (0.8–3.6)	
Residence outside Montevideo§	31 (20)	5 (15)	26 (21)	0.7 (0.2–1.9)	
Chronic medical condition¶	51 (29)	21 (57)	30 (22)	4.7 (2.2–10.2)	
Infection site, nonskin	68 (37)	31 (82)	37 (26)	12.8 (5.2–31.5)	5.1 (1.7–15.1)
Intensive-care unit admission	51 (28)	23 (61)	28 (19)	6.4 (2.9–13.7)	

*CA-MRSA, community-associated methicillin-resistant *Staphylococcus aureus*; CI, confidence interval*.* †Controlling for facility. ‡n = 180; age not available for 2 patients. §n = 159; location of residence not available for 23 patients. ¶n = 175. Chronic conditions: chronic bronchitis, heart disease or stroke, liver or kidney disease, diabetes, HIV, AIDS, or history of immunosuppression or cancer.

**Table 2:** Infection type for 182 hospitalized patients with community-onset CA-MRSA infections, centers A–D, Uruguay, 2003–2004*

Infection type†	Total no. (%) infections, N = 269	Healthcare-onset no. (%), n = 45	Community-onset no. (%), n = 224	p value
Skin (any)	169 (63)	10 (22)	159 (71)	<0.0001
Impetigo	29	2	27	
Foliculitis/pustule	3	0	3	
Abscess	65	3	62	
Furunculosis	9	0	9	
Hidradenitis	1	0	1	
Cellulitis	49	2	47	
Abrasion	4	0	4	
Pressure wound	4	0	4	
Trauma wound	3	3	0	
Burn/necrotic lesion	2	0	2	
Respiratory	36 (13)	17 (38)	19 (9)	<0.0001
Pneumonia‡	34	16	18	
Pleuritis	1	1	0	
Pleural abscess	1	0	1	
Bacteremia	24 (9)	3 (7)	21 (9)	0.78
Surgical site	23 (9)	12 (27)	11 (5)	<0.0001
Organ/space	11 (4)	1 (2)	10 (4)	0.70
Septic arthritis	6	1	5	
Osteomyelitis/myositis	5	0	5	
Indwelling devices	3 (1)	1 (2)	2 (1)	0.42
Catheter infection	1	0	1	
Arteriovenous fistula infection	2	1	1	
Otitis	2 (<1)	1 (2)	1 (<1)	0.31
Cerebral ventriculitis	1 (<1)	0	1 (<1)	1.00

*CA-MRSA, community-associated methicillin-resistant *Staphylococcus aureus*. †Infections are not mutually exclusive (269 infections in 182 patients). ‡Pneumonia or tracheobronchitis.

**Figure 3:**
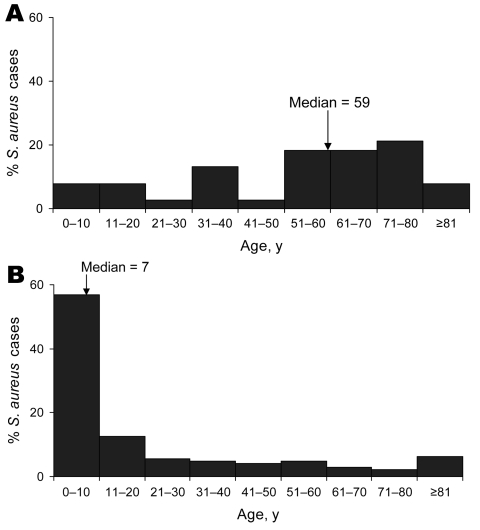
Patient age distribution of A) healthcare-related versus B) community-onset community-associated methicillin-resistant *Staphylococcus aureus* strain type infections, centers A–D, Uruguay, 2003–2004.

Of 182 study patients, 38 (21%) were considered to have healthcare-onset infections. The age distribution of the healthcare-onset and community-onset groups differed with median ages of 59 and 7 years, respectively (Figure 3). Twenty percent of the community-onset group was <2 years of age compared with 3% in the healthcare-onset group. Infection site also differed between the healthcare-onset and community-onset groups; skin infections dominated the community-onset group, whereas most healthcare-onset infections were SSIs or respiratory tract infections (Table 2). ICU admission was more common in the healthcare-onset than in the community-onset group (61% and 19%, respectively, p<0.05) (Table 1).

In multivariable modeling, after the facility was controlled for, age >18 years (odds ratio [OR] 4.8) and non-skin infection sites (OR 5.1) were independently associated with healthcare-onset CA-MRSA strain type infections (Table 1). Effect modification between facility and infection site, facility and age, and infection site and age was not significant.

### Characterization of CA-MRSA Strains

Of 24 isolates selected for molecular typing and toxin testing, 15 (63%) were associated with skin infections, 5 (21%) bloodstream, 3 (13%) respiratory, and 1 (4%) a catheter-site infection. One third of the patients were <18 years of age (3 were <2 years of age), and 50% were female. Nineteen (79%) patients were initially seen at hospitals, 3 (13%) at outpatient centers, and 2 (8%) at a prison. Three (13%) were considered to have healthcare-onset infections.

Twenty-two (92%) of the 24 isolates were of PFGE type USA1100, a community strain (*28*). These 22 isolates contained SCC*mec* type IVc and the PVL locus, and 19 of the 22 had indistinguishable *Sma*I PFGE patterns. The other 3 USA1100 isolates were at least 91% related to the homogeneous group. The 3 healthcare-onset infections were of PFGE type USA1100. The 2 non-USA1100 isolates had microbiologic properties consistent with HA-MRSA belonging to PFGE type USA600 and USA800—one from the respiratory tract and the other from a catheter site. Both of these isolates were from adult patients who were considered to have community-onset infections.

## Discussion

In summary, in at least 1 healthcare facility in Uruguay, CA-MRSA strains appears to be replacing HA-MRSA strains. Transmission of infections caused by CA-MRSA strain types appeared to be occurring in the hospital as well as the community, and patients whose infections developed in the hospital were older and more likely to have non-skin infections than those with community-onset disease.

In the United States, according to the National Nosocomial Infections Surveillance System, MRSA rates have risen during the 1990s and early 2000s (*1*). Klevens et al. (*2*) demonstrated that the antimicrobial resistance profile of ICU MRSA isolates from this surveillance system changed from 1992 through 2003. MRSA consistent with HA-MRSA strain types decreased from 55% in 1992 to 10% in 2003, while CA-MRSA strain types increased from 4% to 14% during the same period. This resistance pattern change was also described in Europe over an 11-year period (1992–2002) in France and over a 4-year period (1995–1998) in Belgium (*29*,*30*). In our study, although the proportion of *S. aureus* infections caused by MRSA remained stable, we observed a dramatic increase (4% to 23%) in the proportion of MRSA consistent with CA-MRSA strain types at 1 large healthcare institution in Uruguay. Whether the proportion of *S. aureus* due to MRSA remains stable or starts to increase over time should be monitored.

Healthcare-onset infections of MRSA are typically caused by HA-MRSA strain types. However, since the emergence of CA-MRSA strains, nosocomial transmission of CA-MRSA strains has been documented. Saiman et al. (*20*) described nosocomial transmission in an outbreak setting among postpartum women in New York City and in San Francisco. Carleton et al. (*31*) found a proportion of nosocomial MRSA isolates with molecular typing consistent with community strains. In Atlanta, during a 7.5-month prospective study in 2004, Seybold et al. (*21*) found that 20% of nosocomial MRSA bloodstream infections were due to USA300, a CA-MRSA strain type. In our study, 38 (21%) hospitalized patients with CA-MRSA strain type infections met our definition for having healthcare-onset infections.

The virulence and transmissibility of CA-MRSA strains in the hospital, compared with that of HA-MRSA strains, are unknown. CA-MRSA strains are typically susceptible to more antimicrobial agents than HA-MRSA strains, but this situation may change as CA-MRSA strains in the healthcare setting are exposed to high antimicrobial selection pressures. In addition, CA-MRSA strains may have opportunities to exchange genetic material with HA-MRSA strains. CA-MRSA strains commonly contain toxin genes such as the PVL toxin gene, which may cause more serious nosocomial infections (*32*). In our study, 22 of the 24 isolates characterized by PCR had the PVL gene.

Typically, patients with CA-MRSA infections have skin and soft tissue infections. However, this might not be the case for healthcare-onset CA-MRSA strain type infections. Davis et al. (*33*) surmise that CA-MRSA strains exposed to healthcare settings may develop characteristics associated with HA-MRSA strains such as decreased accessory gene regulator function, inducible macrolide-lincosamide-streptogrammin resistance, and non–β-lactam resistance patterns. Whether this translates into a clinical picture more consistent with HA-MRSA infections is unclear. According to our findings, healthcare-onset CA-MRSA strain type infections were more likely than community-onset CA-MRSA infections to be non-skin diseases and to occur in older populations, characteristics common to HA-MRSA disease (*34*).

Based on this study and that of Ma et al. (*24*), description of the Uruguayan CA-MRSA outbreak, USA1100 or multilocus sequence type ST30 appears to be the predominant CA-MRSA strain circulating in Uruguay. This strain was identified in Australia, New Zealand, Europe, the United States, and most recently Brazil (*23*,*31*,*35*). In the United States, ST30 accounted for 41% of CA-MRSA infections in 1 study in San Francisco (*31*). However, more recent data from that city suggest replacement of ST30 by ST8 (USA300) (*36*). Whether replacement of this strain in Uruguay will occur, and what its effect on illness and mortality rates would be, remain to be seen.

The emergence of CA-MRSA in Uruguay has been rapid and is associated with significant illness and mortality rates (*37*). Although most CA-MRSA strain type infections in Uruguay are of skin or soft tissue, primary pulmonary disease appearing as necrotizing pneumonia has been described as well as skin or soft tissue infections leading to septic pulmonary embolic events (*38*,*39*). Of note, all isolates tested in our study were susceptible to multiple antimicrobial agents, most importantly TMP/SMX. On the basis of these findings, TMP/SMX may be a viable, cost-effective treatment option for many CA-MRSA infections (*40*).

This study was subject to limitations. Because we based our definition of healthcare-onset CA-MRSA strain type infections on time from hospital admission to culture and no evidence of infection on admission, misclassification was possible. Patients admitted to the hospital for non-MRSA diagnoses may have had CA-MRSA strain skin infections on admission that were not documented or cultured within the first 48 hours. These skin infections would have been differentially misclassified as healthcare-onset and would have diluted the relationship between non-skin infections and healthcare-onset CA-MRSA disease, thereby underestimating our associations. In addition, because electronic laboratory records were unavailable in 3 of the 4 facilities, unrecognized CA-MRSA cases potentially affected the distribution of healthcare and community-onset CA-MRSA strain type cases. However, unless cases were systematically excluded, a selection bias was unlikely to have affected factors associated with healthcare-onset disease. Finally, because we suspected that CA-MRSA strains had entered the healthcare setting, we defined CA-MRSA on the basis of susceptibility patterns rather than epidemiologic risk factors. Without PFGE typing all isolates, it is not possible to determine if all included infections were consistent with known CA-MRSA strain patterns. However, of the 24 isolates that were sent to CDC for characterization and met our definition of CA-MRSA, 22 (92%) were consistent with CA-MRSA strain types.

This study describes characteristics of patients with healthcare-onset CA-MRSA strain type infections but does not identify the risk factors involved in nosocomial transmission such as the presence of indwelling catheters, intubation, certain procedures or surgeries, and specific units within the hospital. In addition, we do not know whether infections were acquired in the hospital or whether patients were colonized before arrival and infections subsequently developed in the hospital.

In conclusion, this study describes CA-MRSA emergence in South America. In addition, similar to what is occurring in other countries, it demonstrates CA-MRSA strain type transmission within healthcare settings. Clinicians should be aware that CA-MRSA strains have entered the healthcare setting and cause skin as well as non-skin infections. These infections may respond to a variety of non–β-lactam antimicrobial agents. Adherence to infection control precautions while one is caring for patients with suspected or confirmed CA-MRSA strain infections is critical for preventing transmission and further penetration of CA-MRSA in healthcare settings.
